# Human dendritic cells activated with MV130 induce Th1, Th17 and IL‐10 responses via RIPK2 and MyD88 signalling pathways

**DOI:** 10.1002/eji.201747024

**Published:** 2017-09-14

**Authors:** Cristina Cirauqui, Cristina Benito‐Villalvilla, Silvia Sánchez‐Ramón, Sofía Sirvent, Carmen M. Diez‐Rivero, Laura Conejero, Paola Brandi, Lourdes Hernández‐Cillero, Juliana Lucía Ochoa, Beatriz Pérez‐Villamil, David Sancho, José Luis Subiza, Oscar Palomares

**Affiliations:** ^1^ Department of Biochemistry and Molecular Biology School of Chemistry Complutense University Madrid Spain; ^2^ Department of Immunology Instituto de Investigación Sanitaria Hospital Clínico San Carlos (IdISSC) Madrid Spain; ^3^ Dpt. of Microbiology I‐Immunology School of Medicine Complutense University of Madrid Madrid Spain; ^4^ Inmunotek S.L. Madrid Spain; ^5^ Centro Nacional de Investigaciones Cardiovasculares Carlos III (CNIC) Madrid Spain; ^6^ Genomics and Microarray Laboratory Department of Medical Oncology Instituto de Investigación Sanitaria Hospital Clínico San Carlos (IdISSC) Madrid Spain

**Keywords:** Dendritic cells (DCs), IL‐10‐producing T cells, Recurrent respiratory tract infections (RRTIs), Th1/Th17 cells, Whole heat‐inactivated polybacterial vaccines

## Abstract

Recurrent respiratory tract infections (RRTIs) are the first leading cause of community‐ and nosocomial‐acquired infections. Antibiotics remain the mainstay of treatment, enhancing the potential to develop antibiotic resistances. Therefore, the development of new alternative approaches to prevent and treat RRTIs is highly demanded. Daily sublingual administration of the whole heat‐inactivated polybacterial preparation (PBP) MV130 significantly reduced the rate of respiratory infections in RRTIs patients, however, the immunological mechanisms of action remain unknown. Herein, we study the capacity of MV130 to immunomodulate the function of human dendritic cells (DCs) as a potential mechanism that contribute to the clinical benefits. We demonstrate that DCs from RRTIs patients and healthy controls display similar *ex vivo* immunological responses to MV130. By combining systems biology and functional immunological approaches we show that MV130 promotes the generation of Th1/Th17 responses via receptor‐interacting serine/threonine‐protein kinase‐2 (RIPK2)‐ and myeloid‐differentiation primary‐response gene‐88 (MyD88)‐mediated signalling pathways under the control of IL‐10. In vivo BALB/c mice sublingually immunized with MV130 display potent systemic Th1/Th17 and IL‐10 responses against related and unrelated antigens. We elucidate immunological mechanisms underlying the potential way of action of MV130, which might help to design alternative treatments in other clinical conditions with high risk of recurrent infections.

## Introduction

Patients suffering from recurrent respiratory tract infections (RRTIs) pose a major health‐care problem with significant morbidity and mortality affecting both children and adults, representing an important economic burden in Europe and USA 1, 2, 3, 4. RRTIs require repeated and prolonged antibiotic cycles, further increasing the risk to develop antibiotic resistances 3, 5, 6, 7. Humankind is entering into a “post‐antibiotic era” with limited treatment options that are causing many deaths yearly due to life‐threatening resistant pathogens 8, 9. Negative factors associated to the overuse of antibiotics also include deleterious effects for the normal microbiome, which favour pathogen invasion and subsequent bacterial and fungal superinfections 10, 11. There is an urgent need for new alternative approaches to antibiotics for the prevention and treatment of RRTIs. Different types of mucosal bacterial vaccines have received a lot of attention over the last years as they significantly reduce the rate of infections in RRTIs patients 3, 4, 12, 13, 14. Bacterial vaccines containing soluble antigens (bacterial lysates) administered through the oral route were initially used to treat and prevent RRTIs 3, 12, 15, 16. More recently, polybacterial preparations (PBP) based on whole inactivated components and delivered through the sublingual route have been also studied as novel alternatives 3, 14. The sublingual PBP MV130 (Bactek) is one of these vaccines. MV130 is composed of different proportions of whole heat‐inactivated Gram‐positive and –negative bacteria often present in the nasal mucosa and frequently involved in upper and lower respiratory infections in Europe (60% *Streptococcus pneumoniae*, 15% *Staphylococcus aureus*, 15% *Staphylococcus epidermidis*, 4% *Klebsiella pneumoniae*, 3% *Moraxella catarrhalis* and 3% *Haemophilus influenzae*) 14. Clinical data showed that MV130 significantly reduced the rate of infections in RRTIs patients, however, the immunological ways of action remain elusive 3, 4, 14. Both specific and nonspecific mechanisms might well be involved in the observed clinical benefits 3, 15, 16, 17. In this study we exclusively focused on the capacity of MV130 to immunomodulate human dendritic cells (DCs) as potential antigen‐independent mechanisms contributing to the clinical benefits. We report that DCs from RRTIs patients and healthy controls display similar *ex vivo* immunological responses to MV130. By combining systems biology and immunological functional experiments, we show that MV130 imprints human DCs with the capacity to generate Th1, Th17 and IL‐10‐producing T cells via receptor‐interacting serine/threonine‐protein kinase‐2 (RIPK2)‐ and myeloid‐differentiation primary‐response gene‐88 (MyD88)‐mediated signalling pathways under the control of IL‐10. In vivo data reveal that sublingual immunization of BALB/c mice promotes potent systemic Th1/Th17 and IL‐10 responses. Our results provide novel insights into the immunomodulatory capacity of MV130 on human DCs as a potential mechanism that in cooperation with antigen‐specific responses might well contribute to the reported clinical benefits in patients suffering from RRTIs. This study also uncovers that the employed methodology constitutes a suitable strategy to elucidate immunological pathways activated by specific PBPs at the molecular level, which might well pave the way to develop more rational patient‐tailored treatments for many other clinical conditions at high risk of recurrent infections.

## Results

### MV130‐activated human DCs produce pro‐inflammatory cytokines with high levels of IL‐10

**Figure 1:**
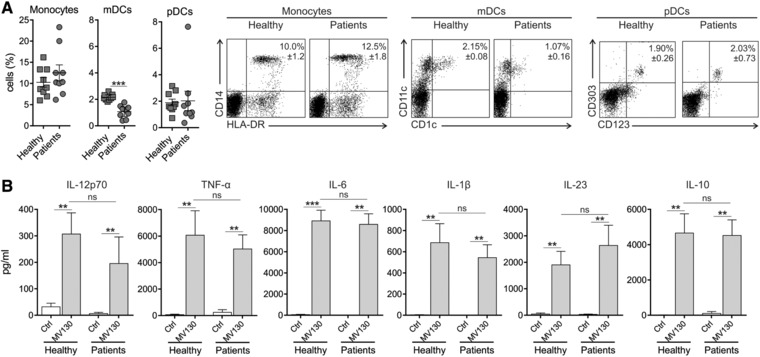
MV130‐activated hmoDCs from healthy subjects and RRTIs patients produce pro‐inflammatory cytokines with high levels of IL‐10. (A) Percentage of monocytes (HLA‐DR^+^ CD14^+^), mDCs (HLA‐DR^+^ CD19^−^ CD1c^+^ CD11c^+^) and pDCs (HLA‐DR^+^ CD123^+^ CD303^+^) in freshly isolated PBMCs from healthy donors (*n* = 9) and RRTI patients (*n* = 9). Data are pooled from three independent experiments with three donor and three patient samples per experiment. Representative dot plots showing the gated cells are displayed on the right side. (B) Cytokines levels in cell‐free supernatants (IL‐12p70, TNF‐α, IL‐6, IL‐β, IL‐23, and IL‐10) quantified by ELISA after stimulation of hmoDCs from healthy subjects (*n* = 9) and patients (*n* = 9) with Ctrl (control containing all excipients except the bacteria) or MV130 for 24 h. All data are represented as mean ± S.E.M. Unpaired (for A) and paired (for B) *t*‐test, *p < 0.05; **p < 0.01; ***p < 0.001.

Nine patients suffering from RRTIs (8 females and 1 male, mean age 52 ± 5; range 35–73) referred to the Clinical Immunology Unit, Hospital Clínico San Carlos of Madrid and nine healthy controls (8 females and 1 male, mean age 35 ± 4; range 22–51) were recruited. All the patients included in the study had suffered RRTIs defined as three or more episodes of upper or lower respiratory tract infections or one pneumonia episode per year for more than one year. None of the patients had previously received MV130 treatment. The detailed clinical features of the patients including main diagnosis and routine therapy are collected in Table 1. First, we quantified and compared the frequency of monocytes and different DC subsets in peripheral blood from healthy subjects and RRTIs patients. The percentage of myeloid DCs (mDCs) in freshly isolated PBMCs was significantly lower in RRTIs patients than in healthy subjects without significant changes in monocytes or plasmacytoid DCs (pDCs) (Fig. 1A). Representative dot‐plots showing the gated cells in each case are also displayed. To assess the capacity of MV130 to immunomodulate the function of human DCs, we generated human monocyte‐derived DCs (hmoDCs) from healthy subjects and RRTIs patients and compared the cytokine signature imprinted by this PBP. MV130**‐** but not control‐treated hmoDCs produced significant increments of the pro‐inflammatory Th1‐ (IL‐12p70 and TNF‐α) and Th17‐driving (IL‐6, IL‐1β, IL‐23) cytokines as well as high levels of IL‐10 without significant differences between DCs from healthy subjects and RRTIs patients (Fig. 1B). Kinetic experiments revealed that IL‐12p35/IL‐12p40, TNF‐α, IL‐6, IL‐1β, IL‐23 and IL‐10 mRNA expression levels were upregulated in MV130‐activated hmoDCs in a time‐dependent manner up to 6 h (Supporting Information Fig. 1). Interestingly, IL‐10 mRNA levels were sustained after 24 h, suggesting that it could be involved in the downregulation of the other assayed pro‐inflammatory cytokines.

**Table 1:** Epidemiological and clinical features of studied patients

Patient number	Sex (F/M)	Age (years)	Main diagnosis	Routine therapy	Infectious history	Frequency (episodes/year)
# 1	F	39	Lymphangioleiomyomatosis. MGUS.	None.	URTIs since infancy. LRTIs. Bilateral pneumonia in 2014. RUTIs since puberty. Bacteriemia in 2014.	5 5 3
# 2	F	74	Recurrent respiratory tract infections. Asthma.	Inhaled fluticasone‐salmeterol.	URTIs. LRTIs.	10 10
# 3	F	49	Selective IgA deficiency. Deficit of antibody production. Celiac disease.	None.	URTIs since infancy. LRTIs since infancy.	7
# 4	M	69	Recurrent respiratory tract infections. MGUS.	Tiotropium, lorazepam, caffeine citrate, nitroglycerin, low dose aspirin.	LRTIs.	10
# 5	F	35	Rinoconjuntivitis‐allergic asthma. Pituitary microadenoma.	Inhaled fluticasone‐salmeterol.	Meningitis at age 3. URTIs. Three previous pneumoniae.	5
# 6	F	64	Rheumatoid arthritis.	Prednisone (10 mg/day).	URTIs. LRTIs. Pneumonia in 2009 y 2013.	3 3
# 7	F	38	Androgen insensitivity syndrome. Low IgM.	Oestradiol/norgestrel.	URTIs. LRTIs. Two pneumoniae episodes in 2013 and 2014.	5 3
# 8	F	34	Common variable immunodeficiency. Bronchiectasis.	IVIG (0,6 g/kg/month).	URTIs. LRTIs.	3 3
# 9	F	57	Bronchoneumopathy chronic obstructive.	None.	URTIs.	3

LRTI: Lower Respiratory Tract Infection (bronchitis and pneumonia); URTI: Upper Respiratory Tract Infection; RUTI: Recurrent Urinary Tract Infection.

MGUS: monoclonal gammopathy of unknown significance. IVIG: intravenous immunoglobulin.

### MV130‐activated hmoDCs promote the generation of Th1, Th17 and IL‐10‐producing T cells

**Figure 2:**
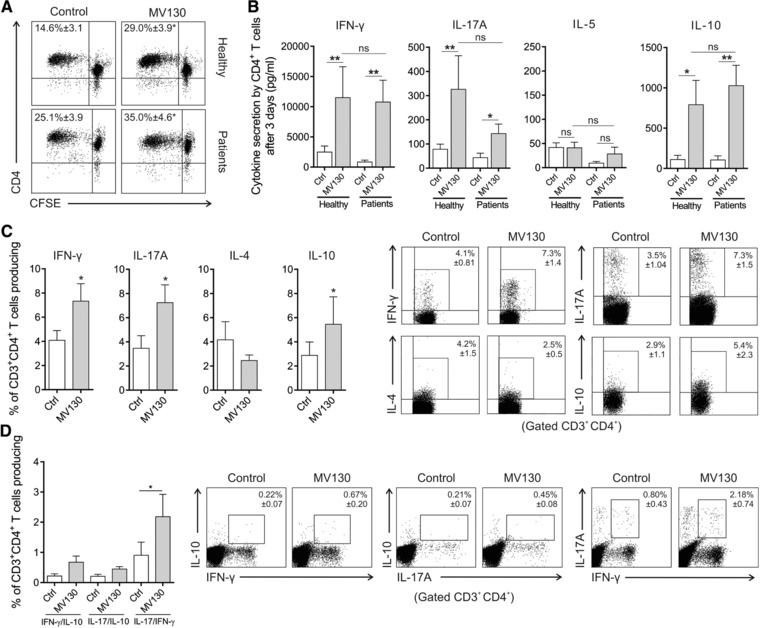
MV130‐activated hmoDCs from healthy donors and RRTIs patients induce T cell proliferation and the generation of Th1, Th17 and IL‐10‐producing T cells. (A) Representative dot plots of proliferating CFSE‐labelled allogeneic naïve CD4^+^ T cells after 3 days of co‐culture with hmoDCs from healthy subjects (*n* = 5) and patients (*n* = 5) in the presence of Ctrl or MV130. The frequency of proliferating cells is displayed inside the plot. (B) ELISA quantification of IFN‐γ, IL‐17A, IL‐5 and IL‐10 cytokines in cell free supernatants produced by allogeneic naïve CD4^+^ T cells primed by Ctrl‐ or MV130‐activated hmoDCs from healthy subjects (n = 9) and patients (*n* = 9) after 3 days. (C) Percentage of CD3^+^CD4^+^ T cells producing IFN‐γ, IL‐17A, IL‐4 and IL‐10 generated after 3 days of co‐culture of Ctrl‐ or MV130‐activated hmoDCs from healthy donors and allogeneic CD4^+^ T cells as determined by intracellular staining (*n* = 4–7). Representative dot plots are shown for each cytokine. (D) Percentage of CD3^+^CD4^+^ T cells simultaneously producing IL‐10 and IFN‐γ, IL‐10 and IL‐17A, or IL‐17A and IFN‐γ after intracellular staining as determined by flow cytometry analysis (n = 5). Panels B, C and D. Data are pooled from 3 to 5 independent experiments. Results are mean ± S.E.M. Wilcoxon‐signed‐rank test *p < 0.05; **p < 0.01.

To assess the capacity of MV130‐activated hmoDCs to polarize T cell responses, we performed co‐culture experiments. MV130‐activated hmoDCs induced a significantly higher percentage of proliferating allogeneic naïve CD4^+^ T cells than control‐treated hmoDCs without significant differences between healthy subjects and RRTIs patients (Fig. 2A). MV130‐activated hmoDCs from both healthy controls and RRTIs patients generated T cells producing significantly higher levels of IFN‐γ, IL‐17A and IL‐10 than control‐treated hmoDCs without IL‐5 production and without significant differences between groups (Fig. 2B). IL‐4 was not detected in cell‐free culture supernatants in any assayed condition (data not shown). Supporting these data, intracellular staining experiments at the single cell level (Fig. 2C) demonstrated that MV130‐activated hmoDCs generate Th1/Th17 and IL‐10‐producing T cells without significant induction of Th2 responses. Interestingly, double intracellular staining experiments showed that a significantly higher population of CD4^+^ T cells coexpressing IFN‐γ and IL‐17A was generated by MV130‐activated DCs than control. However, we did not detect CD4^+^ T cells simultaneously producing IFN‐γ and IL‐10 or IL‐17A and IL‐10 (Fig. 2D).

### MV130‐activated total blood DCs from healthy and RRTIs patients show similar functional responses

**Figure 3:**
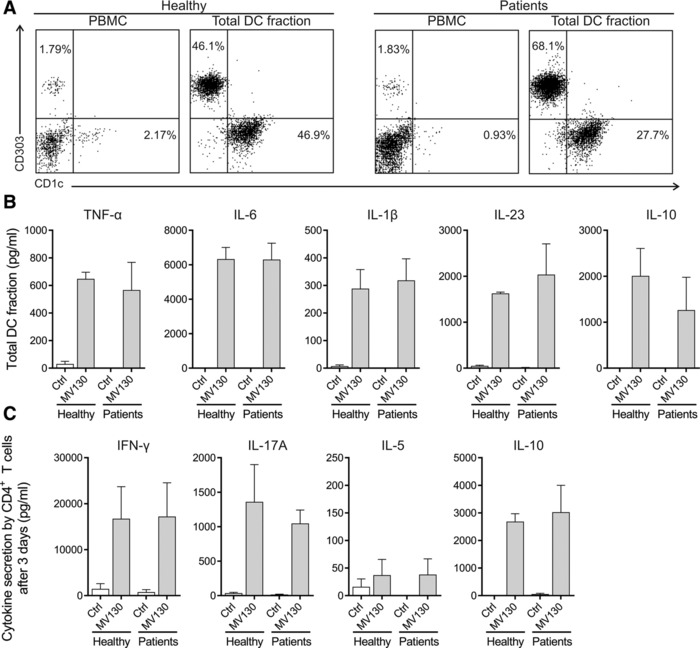
MV130‐activated total blood DCs from healthy subjects and RRTIs patients produce pro‐ and anti‐inflammatory cytokines and induce Th1, Th17 and IL‐10‐producing T cells. (A) Representative dot plots of the different DC subsets contained in PBMCs from healthy subjects and RRTIs patients and the obtained enriched total DC fraction in each case: pDCs (HLA‐DR^+^ CD19^−^ CD303^+^); mDCs (HLA‐DR^+^ CD19^−^ CD1c^+^). The percentage for each DC subset is displayed inside the quadrants. Data are representative of three independent experiments. (B) Cytokine production by the enriched total DC fraction from healthy individuals and patients after 24 h of stimulation with Ctrl or MV130 quantified by ELISA. Data are pooled from three independent experiments (C) Cytokines produced by allogeneic naïve CD4^+^ T cells primed by control‐ or MV130‐activated total DCs from healthy subjects and patients after 3 days quantified by ELISA. Results are the mean ± S.E.M. of three independent experiments.

We obtained an enriched fraction of total blood DCs containing both mDCs and pDCs from PBMCs of healthy subjects and patients suffering from RRTIs (Fig. 3A). The total blood DC fraction activated with MV130 produced higher levels of TNF‐α, IL‐6, IL‐1β, IL‐23 and IL‐10 than control‐stimulated cells (Fig. 3B). Similar responses were observed in total blood DCs from healthy subjects and RRTIs patients for the assayed cytokines. Co‐culture experiments demonstrated that MV130‐ but not control‐treated total blood DCs from both groups also generated T cells producing high levels of IFN‐γ, IL‐17A and IL‐10 but not IL‐5 (Fig. 3C).

**Figure 4:**
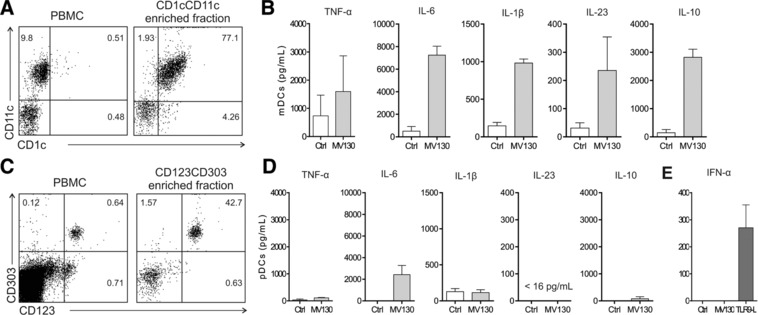
Enriched mDCs produce proinflammatory cytokines and IL‐10 after MV130 stimulation whereas enriched pDCs only IL‐6. (A) Representative dot plots for mDCs contained in PBMCs and the obtained enriched mDCs (HLA‐DR^+^ CD19^−^ CD1c^+^ CD11c^+^). (B) Cytokine production by the enriched mDCs after 24 h of stimulation with Ctrl or MV130 quantified by ELISA. (C) Representative dot plots for pDCs contained in PBMCs and the obtained enriched pDCs (HLA‐DR^+^ CD123^+^ CD303^+^). (D) Cytokines produced by the enriched pDCs after 24 h of stimulation with Ctrl or MV130 quantified by ELISA. Panels B and D: Data are pooled from 3 independent experiments. (E) IFN‐α production by enriched pDCs stimulated for 24 h with Ctrl, MV130 or TLR9‐ligand as positive control. Results are the mean ± S.E.M. of three independent experiments.

To further assess the potential contribution of mDCs and pDCs to the observed effects we obtained enriched fractions of both DCs subsets from healthy donors (Fig. 4A and C) to assess cytokine production after MV130 stimulation, including IFN‐α responses in pDCs. Enriched mDCs produced higher levels of all the assayed proinflammatory cytokines and IL‐10 after MV130 stimulation than control (Fig. 4B). In contrast, pDCs only produced IL‐6 after MV130 stimulation (Fig. 4D). IFN‐α production was only detected after stimulation of enriched pDCs with TLR9‐L as positive control but not after MV130 stimulation (Fig. 4E). Collectively, these data indicate that mDCs are the main responder DC subset after MV130 stimulation and suggest that although RRTI patients display lower percentages of circulating mDCs than healthy donors, they are functional and might well play an important role in the MV130 treatment for these patients.

### Uncovering MV130‐activated signalling pathways in human DCs by global comparative transcriptomics

**Figure 5:**
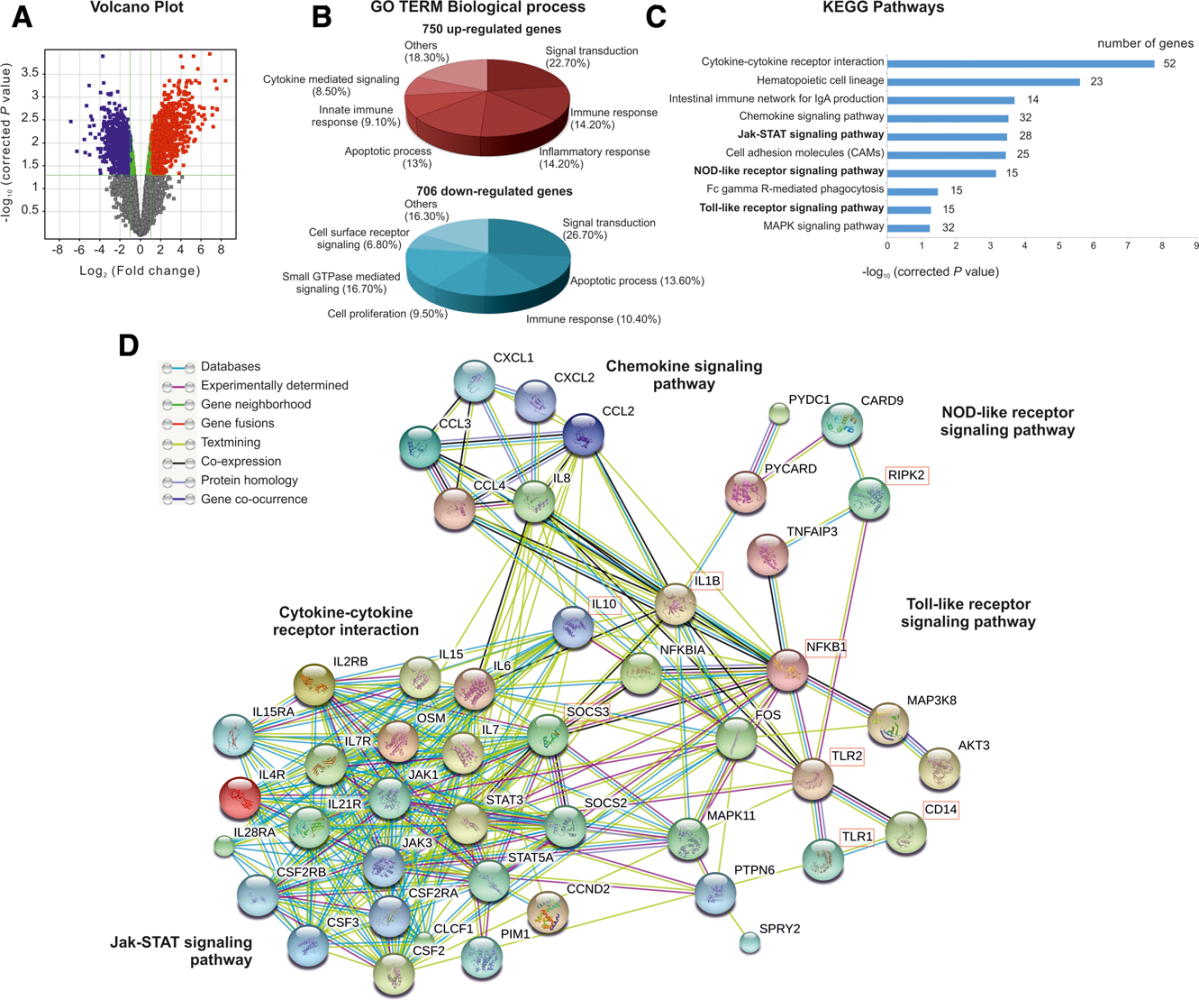
Global comparative transcriptome analysis by DNA microarrays of hmoDCs treated with control or MV130 for 24 h. (A) The Volcano plot depicts −log_10_ of corrected *p* value versus log_2_ of fold‐change (FC) for each gene. Moderate *t*‐test analysis was used for *n* = 4 independent experiments to find 1456 genes differentially expressed (FC > 2 and *p* < 0.05) between control and treated samples. Up‐ and down‐regulated genes are highlighted in red and blue, respectively. (B) GO term analysis using GeneCodis software was performed for the up‐ and down‐regulated genes. Altered GO terms at *p* < 0.05 and > 10 genes are displayed in red and blue chart diagrams for up‐ and down‐regulated genes, respectively. (C) KEGG analysis of the 10 altered (*p* < 0.06 and > 10 genes) immunoregulatory pathways analyzed with DAVID software according to the −log10 of corrected *p* value and the number of genes involved in each pathway. (D) Protein interaction network using as input the 49 non‐redundant genes from JAK‐STAT, NLR and TLR signalling pathways with the STRING program at confidence of ≥ 0.7. The main clusters of associated genes identified by the predicted network are indicated. Key molecules for each cluster are highlighted. The connected lines represent the associations according to the color code indicated in the figure. Data are pooled from 4 independent experiments.

To gain insight into the underlying molecular mechanisms involved in the effects imprinted by MV130 in human DCs, we performed microarray genome wide expression analysis between MV130‐ and control‐treated hmoDCs. Considering that MV130 imprints the same responses in human DCs from healthy and RRTIs patients, we employed hmoDCs from healthy donors. The 1456 genes were differentially expressed (FC > 2; p* *< 0.05) in MV130‐treated hmoDCs compared to control (Fig. 5A). Functional enrichment analysis for the 750 up‐ and 706 down‐regulated genes by the gene ontology term biological processes showed around 50% of them clustered in signal transduction, immune responses and apoptotic biological processes (Fig. 5B). The rest of up‐regulated genes were mainly gathered in biological processes related with immune system functions whereas down‐regulated genes in cell proliferation and signalling (Fig. 5B). Functional gene set enrichment analysis of the 1,456 genes by Kyoto Encyclopedia of Genes and Genomes (KEGG) for pathways allowed the identification of 10 immunoregulatory signalling pathways differentially activated in MV130‐treated hmoDCs (Fig. 5C). A predicted network of interactions using as input the 49 non‐redundant genes clustered in the Janus kinase‐signal transducer and activator of transcription (JAK‐STAT), nucleotide‐binding oligomerization domain‐like receptors (NLR) and Toll‐like receptor (TLR) signalling pathways is shown (Fig. 5D). The predicted network suggested that MV130 triggers TLRs and NLRs in human DCs leading to the activation of NF‐κB and the production of pro‐inflammatory cytokines, chemokines and IL‐10. The interaction of cytokines and chemokines with cognate receptors integrates JAK‐STAT downstream signalling pathways that also could contribute to finely tune immune responses in human DCs 18, 19, 20. Interestingly, a central role for IL‐10 in the control of potential excessive pro‐inflammatory responses, for example, by inducing SOCS genes is also predicted (Fig. 5D).

### RIPK2‐, MyD88‐mediated pathways and IL‐10 drive immune responses in MV130‐activated human DCs

**Figure 6:**
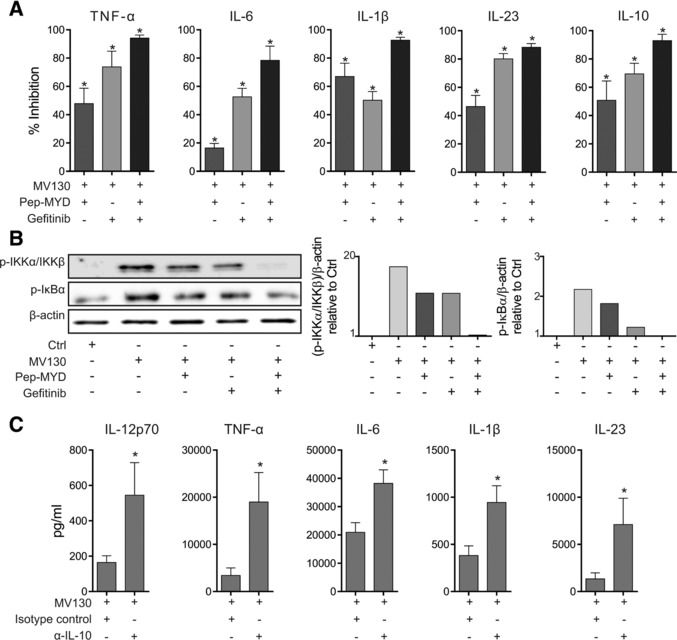
MV130 immunomodulates human DCs’ function by mechanisms depending on NLR/RIPK2‐ and TLR/MyD88‐mediated signaling pathways under the control of IL‐10. (A) The graphs display the percentage of inhibition of TNF‐α, IL‐6, IL‐1β, IL‐23 and IL‐10 production by MV130‐treated hmoDCs from healthy donors by the indicated inhibitors (Pepinh‐MYD and/or Gefitinb) related to vehicle controls (Pepinh‐Control and/or DMSO). Results are pooled from 6–8 independent experiments. Mean ± S.E.M. is shown. Wilcoxon‐signed‐rank test *p < 0.05. (B) Western blot analysis of protein extracts from hmoDCs stimulated for 30 min under the indicated conditions (Ctrl, MV130, Pepinh‐MYD or Gefitinb) and quantification of the reactive phosphorylated bands by scanning densitometry. Graphs in the right side represent the quantification of the corresponding reactive bands (phospho‐IKKα/IKKβ (Ser176/Ser177) and phospho‐IkBα (Ser32/36)) with respect to the β‐actin and relative to Ctrl‐treated condition for each case. One representative example out of 3 is shown. (C) Increase of IL‐12p70, TNF‐α, IL‐6, IL‐β and IL‐23 cytokine production by MV130‐activated hmoDCs in the presence of specific blocking antibodies against IL‐10 (α‐IL‐10) with respect to the levels in the presence of isotype control. Results are pooled from six independent experiments. Mean ± S.E.M. is shown. Wilcoxon‐ signed‐rank test *p < 0.05.

To validate the pathways identified by global comparative transcriptomic analysis, we performed inhibition experiments using pepinh‐MYD (an intracellular peptide blocking MyD88‐mediated signalling pathways coupled to TLRs) and Gefitinib (a selective pharmacological inhibitor of RIPK2, the adaptor protein for NOD1/NOD2‐mediated signalling pathways). The production of TNF‐α, IL‐6, IL‐1β, IL‐23 and IL‐10 by MV130‐activated hmoDCs was significantly impaired (inhibition up to 95%) by simultaneous preincubation with pepinh‐MYD and Gefitinib (Fig. 6A). The individual contribution of TLRs and NLRs for each cytokine is also displayed. MV130 activated NF‐κB in human DCs as determined by the phosphorylation of IKKα/IKKβ at Ser176/Ser177 and IκBα at Ser32/36, which was totally abolished by simultaneous inhibition with pepinh‐MYD and Gefitinib (Fig. 6B). Anti‐IL‐10 blocking antibodies (α‐IL‐10) significantly increased the production of IL‐12p70, TNF‐α, IL‐6, IL‐1β and IL‐23 in MV130‐activated hmoDCs at the protein level (Fig. 6C).

### MV130 sublingual immunization induces Th1/Th17 and IL‐10 mediated responses in mice

**Figure 7:**
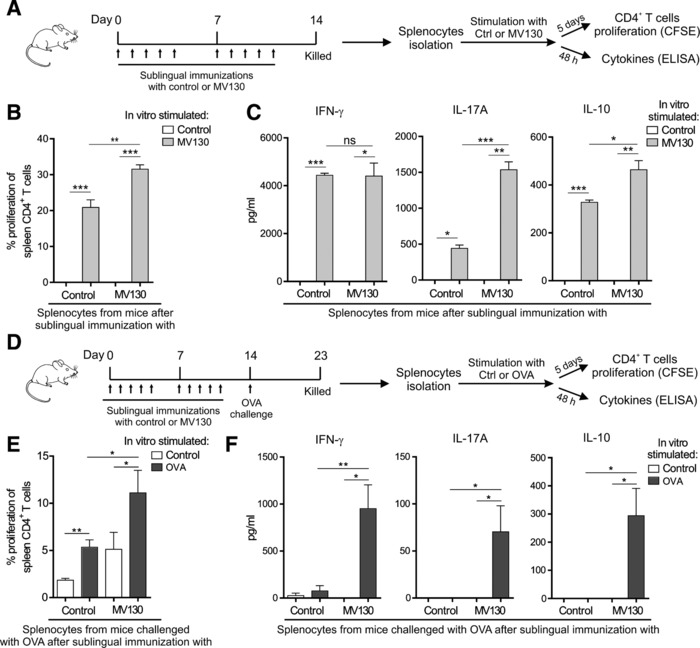
Induction of Th1, Th17 and IL‐10 immune responses after in vivo MV130 sublingual immunization of BALB/c mice. (A) Scheme of the sublingual immunization protocol and analysis of induced systemic responses. (B) Proliferation of CFSE‐labelled CD4^+^ T cells from splenocytes isolated from mice immunized sublingually with MV130 or control after in vitro stimulation with MV130 or control. (C) Cytokine production (IFN‐γ, IL‐17A and IL‐10) by splenocytes isolated from mice immunized sublingually with MV130 or control and stimulated in vitro with MV130 or control. (D) Scheme of the immunization/challenge protocol followed to assess immune responses against the unrelated antigen OVA. (E) Proliferation of CFSE‐labelled CD4^+^ T cells from splenocytes isolated from the indicated mice after in vitro stimulation with OVA or control. (C) Cytokine production by the indicated splenocytes stimulated in vitro with OVA or control. Results are mean ± S.E.M. of *n* = 6 (B), *n* = 3 (C), *n* = 8 (E) and *n* = 6 (F) from two independent experiments. Ctrl, control containing all excipients except the bacteria; MV130, PBP Bactek. Unpaired or paired *t* test, *p < 0.05; **p < 0.01; ***p < 0.001.

To study the in vivo relevance of our findings BALB/c mice were sublingually immunized with MV130 or control excipients as shown in Fig. 7A. Systemic responses were assessed after in vitro stimulation of splenocytes isolated from MV130 or control immunized mice as indicated in Fig. 7A. Spleen CD4^+^ T cells from both MV130‐ or control‐immunized mice showed significantly higher proliferation rates when stimulated in vitro with MV130 than control (Fig. 7B). Interestingly, spleen CD4^+^ T cells from mice immunized with MV130 displayed significantly higher proliferation rates than those from control immunized mice after MV130 in vitro stimulation (Fig. 7B). Splenocytes from both groups stimulated in vitro with MV130 produced significantly higher levels of IFN‐γ, IL‐17A and IL‐10 than those stimulated with control excipients (Fig. 7C). Interestingly, the levels of IL‐17A and IL‐10 produced by splenocytes from mice sublingually immunized with MV130 were significantly higher than those produced by splenocytes from control mice after in vitro MV130 stimulation (Fig. 7C). The levels of IFN‐γ were also significantly high but without differences between groups (Fig. 7C). We did not detect production of IL‐5 in any of the assayed conditions (data not shown).

To assess the capacity of MV130 to enhance systemic immune responses also against unrelated antigens, mice sublingually immunized with MV130 or excipients as control were challenged in vivo with OVA and splenocytes collected to assess in vitro OVA‐specific immune responses as shown in Fig. 7D. Splenocytes from mice that were sublingually immunized with MV130 and subsequently challenged in vivo with OVA displayed significantly higher proliferation rates after in vitro stimulation with OVA than those from mice sublingually immunized with control excipients (Fig. 7E). Remarkably, OVA‐specific T cells from mice sublingually immunized with MV130 produced significantly higher levels of IFN‐γ, IL‐17 and IL‐10 than those from the control group (Fig. 7F). We did not detect significant differences in OVA‐specific IgG1 or IgG2a in the assayed conditions (data not shown). Collectively, these data showed that sublingual immunization with MV130 also significantly enhances Th1, Th17 and IL‐10 immune responses against the unrelated antigen OVA.

## Discussion

In this preclinical study, we combined systems biology and functional immunological approaches to demonstrate that MV130, a sublingual PBP to treat and prevent RRTIs, imprints human DCs with the capacity to generate Th1, Th17 and IL‐10‐producing T cells via RIPK2‐ and MyD88‐mediated signalling pathways under the control of IL‐10. We also showed that sublingual immunization of BALB/c mice with MV130 induces systemic Th1, Th17 and IL‐10 responses in vivo against related and unrelated antigens. Our data uncover the immunological mechanisms by which this specific PBP immunomodulates the function of human DCs, which in cooperation with antigen‐specific responses might well also contribute to the reported clinical benefits in patients suffering from RRTIs. The understanding of these immunological mechanisms might well help to the designing of rational patient‐tailored novel bacterial vaccines in other clinical conditions at high risk of recurrent infections.

RRTIs are the first leading cause of community‐ and nosocomial‐acquired infections. Antibiotics remain the mainstay of treatment for RRTIs, but repeated and prolonged antibiotic cycles in these patients enhance the potential to develop antibiotic resistances. That is why new alternative approaches to prevent and treat RRTIs have brought a lot of attention over the last years. At this regard, bacterial lysates from different species administered through the oral route were initially employed and showed clinical efficacy in the reduction of respiratory infections 3, 12, 13. Compelling experimental evidence demonstrated that whole inactivated bacteria induce more potent and durable responses than soluble bacterial antigens or lysates 21, 22. Nowadays, it is well‐recognized that sublingual immunization protects bacterial components from gastrointestinal degradation and promotes long‐lasting immune responses locally and in distant target tissues 23, 24, 25, 26. MV130 (Bactek), is a novel sublingual polybacterial vaccine that encompasses both features. It is composed by whole heat‐inactivated bacteria (90% Gram‐positive and 10% Gram‐negative) causing the majority of respiratory bacterial infections in Europe and it is delivered through the sublingual route 3, 4. A pilot clinical study showed that daily sublingual administration of MV130 significantly reduced the rate of respiratory infections in RRTIs patients 14, however, the immunological mechanisms of action remained unknown. We studied the molecular pathways underlying the potential capacity of MV130 to immunomodulate the function of human DCs as an alternative way of action of these preparations. The clinical benefits reported after administration of mucosal polyvalent bacterial vaccines via the sublingual route likely involve both antigen‐specific and nonspecific immunological and immunomodulatory effects 16, 17. A dense network of functional DCs playing a key role in the initiation and maintenance of proper immune responses is located beneath the human sublingual epithelium and tonsils 27, 28, 29, 30. Therefore, we focused on the capacity of MV130 to immunomodulate the function of DCs from patients suffering from RRTIs and healthy subjects by analysing the immunological mechanism imprinted by this PBP at the molecular level.

Our data revealed that MV130 promotes a potent pro‐inflammatory response, inducing the secretion of cytokines to bias toward Th1 and Th17 responses, which might contribute to enhance immune responses against intracellular and extracellular pathogens, respectively 31, 32, 33, 34. On the other hand, MV130 also induces the production of high levels of IL‐10 by DCs and the generation of IL‐10‐producing T cells, which is essential to avoid excessive deleterious responses, to enhance pathogen clearance and to keep tissue homeostasis 35, 36, 37. Although the patients suffering from RRTIs included in this study display fewer numbers of mDCs than healthy subjects, MV130 was able to immunomodulate DCs from both groups in a similar manner. Our data demonstrating that BALB/c mice daily sublingually immunized with MV130 for two weeks display potent systemic Th1, Th17 and IL‐10 responses support the in vivo relevance of our findings. Remarkably, MV130 was also able to enhance in vivo immune responses against unrelated antigens such as OVA, suggesting the potential capacity of this polybacterial preparation to confer protection not only against the components included in the vaccine but also against a broad range of different potential pathogens. Global comparative transcriptome analysis by using DNA microarrays combined with functional immunological in vitro experiments allowed us to uncover that MV130 imprints human DCs with the capacity to generate Th1/Th17 and IL‐10‐producing T cells by mechanisms depending on RIPK2 and MyD88, key signalling and adaptor molecules for NLRs and TLRs, respectively 38, 39. DCs are equipped with a large number of pattern recognition receptors (PRRs) that enable them to link innate and adaptive immune responses 28, 40, 41, 42, 43. Among them, NLRs and TLRs play essential roles in regulating human DC function including their capacity to polarize T cell responses 38, 39, 44, 45, 46. Interestingly, our inhibition experiments using anti‐IL‐10 blocking antibodies established the important role played by IL‐10 produced by MV130‐activated DCs in the control of excessive immune responses. IL‐10 drives key immune regulatory functions by regulating different suppressive mechanisms 34, 35, 47, 48, 49.

Collectively, we demonstrated that MV130, a sublingual whole inactivated PBP that prevents infections in patients suffering from RRTIs, directly acts on human DCs promoting the generation of Th1/Th17 responses by mechanisms depending on NLR‐ and TLR‐mediated signalling pathways under the control of IL‐10. We have recently shown that MV140, a sublingual PBP formulated with a different composition of whole‐inactivated bacteria to treat recurrent urinary tract infections, also generates Th1, Th17 and IL‐10 immune responses 50. Interestingly, the mechanisms underlying the effects of MV140 (75% Gram‐negative/25% Gram‐positive) depend on spleen tyrosine kinase (Syk)‐mediated signalling pathways in cooperation with MyD88 50, which are different to those reported in this study for MV130 (90% Gram‐positive/10% Gram‐negative). Although TLRs contribute to the final outcomes in both cases, the relative contribution in MV130 and MV140 is also different (Supporting Information Fig. 2). As shown in this figure, the contribution of TLRs to the induction of all the assayed cytokines, except for IL‐6, was significantly higher for MV130 than MV140 in human DCs. Overall, our data uncover that although the clinical outcomes (significant reduction of recurrent infections in different clinical settings) seem to be similar for different PBPs, the molecular mechanisms leading to the immunomodulation and phenotypes underpinning clinical benefits might completely differ depending on the specific bacterial components in each PBP. The translation of this concept to clinical practice might well pave the way to develop more rational patient‐tailored novel vaccines for many other clinical conditions at high risk of recurrent infections, which might well also contribute to combat the spread of antibiotic resistant infections.

## Materials and methods

### Media and reagents

RPMI 1640 (Lonza) supplemented with 10% fetal‐bovine‐serum, 100 μg/mL normocin, 50 μg/mL penicillin‐streptomycin, 1% non‐essential aminoacids, 1% MEM‐vitamins and 1 mM sodium pyruvate (cRPMI). PBP MV130 (Bactek)composed of heat‐inactivated bacteria (60% *S. pneumoniae*, 15% *S. aureus*, 15% *S. epidermidis*, 4% *K. pneumoniae*, 3% *M. catarrhalis* and 3% *H. influenzae*), PBP MV140 (Uromune) composed of heat‐inactivated bacteria (*Escherichia coli*, *Proteus vulgaris*, *Klebsiella pneumoniae* and *Enterococcus faecalis*, 25% each) and control (all the excipients without bacteria) were from Inmunotek S.L. Type B TLR9‐ligand (ODN 2006), inhibitors for MyD88 (Pepinh‐MYD), RIPK2 (Gefitinib) from InvivoGen and neutralizing anti‐IL‐10 (clone JES3‐9D7, Biolegend) were used.

### Patients

The clinical features of the 9 patients suffering from RRTIs included in this study are summarized in Table 1. The study was approved by the Ethics Committee of Hospital Clínico San Carlos, Madrid, Spain (C.P.‐C.I.16/191‐E_TFG). Written informed consent was obtained from all the subjects included in the study.

### PBMC, human‐monocyte derived dendritic cells (hmoDCs), naïve CD4^+^ T cells, total DC fraction and cell cultures

PBMC from healthy donors and patients were isolated by Ficoll‐Paque Plus (GE‐Healthcare) density gradient centrifugation from heparinized blood as described 40, 43. Monocytes were isolated from PBMC with anti‐human CD14 microbeads in autoMACS (Miltenyi‐Biotec) and cultured in RPMI medium with recombinant human GM‐CSF and IL‐4 (100 ng/mL each; PeproTech). After 6 days, immature hmoDCs were harvested and phenotypically characterized by flow cytometry. Naïve CD4^+^ T cells, total DC fraction, enriched fractions of mDCs and pDCs were isolated with “Naïve CD4^+^ T Cell”, “Blood DCs II”, CD1c (BDCA‐1)^+^ and CD304 (BDCA‐4/Neuropilin‐1) Isolation Kits (Miltenyi‐Biotec), respectively. Immature hmoDCs, total blood DC, enriched mDCs and pDCs (10^6^ cells/mL) were stimulated with control or MV130 (10^7^ bact./mL) for 24 h. The used dose of MV130 was selected based on previous dose‐depending titration experiments to be optimal for the production of all the assayed cytokines. IL‐12p70, TNF‐α, IL‐6, IL‐1β, IL‐23 and IL‐10 were quantified by ELISA. For inhibition experiments, hmoDCs were preincubated for 6 h with Pepinh‐MYD (50 μM), 30 min with Gefitinb (5 μM) or 1 h with anti‐IL‐10 (2.5 μg/mL) (or their corresponding controls) prior to stimulation. HmoDCs or blood DC fraction were co‐cultured with purified allogeneic naïve CD4^+^ T cells (1:5 DC:T cell ratio) for 3 days in the presence of the control or MV130 (10^7^ bact./mL) as described 27, 28. IFN‐γ, IL‐17A, IL‐5 and IL‐10 were quantified by ELISA. For intracellular staining, the primed CD4^+^ T cells were washed and re‐stimulated with 25 ng/mL PMA plus 1 μg/mL ionomycin for 6 h and Brefeldin A (10 μg/mL) for last 4 h. Cells were fixed, permeabilized and stained for IFN‐γ, IL‐17A, IL‐4 and IL‐10. Purified allogeneic naïve CD4^+^ T cells were labelled with CFSE (Molecular Probes) prior to co‐cultures and proliferation assessed in CFSE dilution experiments by flow cytometry.

### Flow cytometry

Flow cytometry was performed at the UCM Cytometry and Fluorescence Microscopy Unit. Cells were washed with PBS/EDTA 2 mM/0.5% BSA and stained for 15 min at room temperature with the fluorescence‐labelled antibodies or corresponding isotype controls. The following anti‐human monoclonal antibodies (mAbs) were used for flow cytometry: fluorescein isothiocyanate (FITC)‐conjugated anti‐CD1c, anti‐HLA‐DR, anti‐CD123; allophycocyanin (APC)‐conjugated anti‐HLA‐DR; phycoerythrin (PE)‐conjugated anti‐CD11c and anti‐CD303; peridinin‐chlorophyll‐protein (PerCP)‐conjugated anti‐CD14 and anti‐CD4 (Myltenyi Biotec). APC‐conjugated anti‐CD3; Alexa Fluor 488‐conjugated anti‐IFN‐γ and anti‐IL‐4 (BD Pharmigen). PE‐conjugated anti‐IL‐10 and anti‐IL17A; PE/Cy7‐conjugated anti‐CD19 and Alexa488‐conjugated anti‐IL‐17A (BioLegend). For each staining, the corresponding isotype controls (IgG2A‐FITC, IgG1‐PE, IgG2A‐PerCP or IgG1‐APC) were also assayed.

### Cytokine quantification

IL‐12p70, TNF‐α, IL‐6, IL‐1β, IL‐10, IL‐5, and IFN‐γ in cell‐free supernatants were quantified using specific ELISA cytokine kits (BD Biosciences); IL‐23 using Human IL‐23 ELISA Ready‐SET‐Go!® (e‐Biosciences), IL‐17A using Quantikine Elisa Kit (RD Systems) and IFN‐α using Verikin^TM^ ELISA kit (PBL assay science). In all cases, manufacturer's instructions of the specific kits were followed with minor modifications. Briefly, the catching mAbs were coated onto microtiter plates that were blocked with assay diluent (PBS 1X with 5% FBS) and incubated with the standards or samples. After washing, biotinylated anti‐human mAbs and streptavidin‐labelled peroxidase were added to detect bound cytokines. After washing, chromogenic substrate (0.63 mg/mL OPD, 0.03% H_2_O_2_ in 1 M sodium citrate) was added. The colour reaction was stopped by adding 3N H_2_SO_4_ and the OD values were measured at 492 or 450 nm according to each kit.

### DNA microarray analysis

RNA was extracted using TRIZOL (Invitrogen) from hmoDCs from healthy subjects stimulated with control or MV130 for 24 h. RNA quality was measured with Agilent Bioanalyzer 2100 (Agilent technologies) and only good quality samples, RIN (RNA Integrity Number) higher than 7.5, were selected for the analysis. Agilent G4851B (8 × 60K) microarrays were used to analyze gene expression in 4 control‐ and 4 MV130‐treated hmoDCs from healthy donors. Agilent recommended protocol “Low Input Quick Amp Labeling Kit, One‐Color (G4140‐90040)” was followed. Briefly, 200 ng of total RNA together with the appropriate RNA spike‐in (RNA Spike‐In Kit, One‐Color Agilent p/n 5188–5282) were subjected to reversed transcription in the presence of T7‐primer, dNTPs, first strand buffer and enzymes for 2 h at 40°C. Afterwards cRNA was synthesized and Cy3‐labelled in the presence of transcription buffer, NTPs and T7‐RNA Polymerase. Labelled cRNA was purified using Qiagen columns (RNeasy Mini Kit). RNA concentration was measured using the NanoDrop ND‐1000 spectrophotometer and the yield and specific activity were determined. 600 ng of cRNA were hybridized to the microarray for 17 h at 65°C. Fluorescence was measured using Agilent microarray scanner and Feature Extraction software. Quality Control Report and spikes‐in were used to discard the microarrays that did not fulfill good quality criteria. Gene Spring GX software (Agilent) was employed for data normalization (75% percentile shift) and analysis. From the original 60 K features microarray, 19 893 spots without flags expressed in 90% of the samples were used for the following statistical analysis. Differential gene expression assessment was carried out using Moderate T test and Benjamini‐Hochberg false discovery rate for multiple correction. Selected genes showed a FC > 2 and a p < 0.05. Transcript profiling: [ArrayExpress # E‐MTAB‐5259].

Gene set enrichment analysis was carried out by using Gene Codis software web‐based tool (http://genecodis.cnb.csic.es) 51, 52 DAVID Bionformatics Resources 6.8 software (https://david.ncifcrf.gov/) 53. Protein interaction analysis was performed with the STRING 10.0 (known and predicted protein‐protein interactions) free program (http://string-db.org/).

### Western blot

Freshly isolated hmoDCs were previously pre‐incubated with Pepinh‐MYD or Pepinh‐control for 6 h and with Gefitinib or DMSO for 30 min before the stimulation with control or MV130 for 30 min. Cell lysis was carried out with PBS/Triton 1% in the presence of 1 mM PMSF (Sigma), 1 μg/mL Leupeptin (Bachem) and Aprotinin (Roche) for 30 min at 4 °C with vortex every 10 min. Lysates were clarified by centrifugation at 10 000 × *g* for 15 min at 4°C. Protein quantification was performed with Micro BCA Protein Assay Kit (Pierce) according to the manufacturer's instructions and samples with equal amounts of total protein were resolved in 10% SDS‐PAGE. Proteins were then transferred to nitrocellulose membranes.

The membranes were incubated with the following antibodies: anti‐human phospho‐IkBα (Ser32/36) (1/1000, Cell signalling), anti‐human phospho‐IKKα/IKKβ (Ser176/Ser177) (1/1000, Cell signalling), and anti‐human β‐actin (1/5000, Sigma) as primary antibodies and Goat anti‐mouse or anti‐rabbit conjugated with horseradish peroxidase (1/2500, Pierce) as secondary antibodies. Reactive bands were visualized by using ECL chemiluminescence system (BioRad). Optical density of the reactive bands was quantified with Fujifilm multigauge software and values expressed relative to β‐actin loading control.

### RNA extraction, cDNA synthesis, and quantitative real‐time RT‐PCR

RNA was isolated from harvested cells using an RNeasy mini kit (Qiagen) and cDNA generated using a PrimeScript RT reagent Kit (Takara) according to manufacturers’ instructions. Real‐time quantitative PCR was performed on cDNA with FastStart Universal SYBR Green Master (Rox) (Roche). The sequences of the used pair primers were: Elongation Factor 1α (forward, CTGAACCATCCAAT; reverse, GCCGTGTGGCAATCCAAT), IL‐12p35 (forward, ATGATGGCCCTGTGCCTTAGT; reverse, TGCCTCTTAGGATCCATCAGAAG), IL‐12p40 (forward, GCATCTGTGCCCTGCAGTTA; reverse, CTTATTATCTTCCACTTTTCCTCCAAA), IL‐6 (forward, GGTACATCCTCGACGGCATCT; reverse, AGTGCCTCTTTGCTGCTTTCAC), IL‐1β (forward, TTTTTGCTGTGAGTCCCGGAG; reverse, TTCGACACATGGGATAACGAGG), IL‐23 (forward, TCCCCATATCCAGTGTGGAGAT; reverse, GTGGATCCTTTGCAAGC) and IL‐10 (forward, GTGATGCCCCAAGCTGAGA; reverse, CACGGCCTTGCTCTTGTTTT). Samples were run on a real‐time PCR system (ABI Prism 7900 HT; Applied Biosystems). Data were normalized to Elongation Factor 1α (EF1α) and displayed as 2^−ΔCT^ values multiplied by 10^4^. ΔCT was defined as the difference between the cycle threshold value for the gene of interest and Elongation factor 1α.

### Mice experiments

Animal experiments were approved by the Ethics Committee of Hospital Clínico San Carlos and Centro Nacional de Investigaciones Cardiovasculares Carlos III (CNIC) and performed in accordance with the Spanish national and international/EU legislation regulated by D.C.86/609/CEE; RD 1201/2005. BALB/c mice (6‐weeks‐old) were sublingually immunized for 5 consecutive days during two weeks with MV130 (10^9^ bact./mL) or control and sacrificed 2 days after the last immunization. For the experiments to assess potential antigen‐independent effects, mice were intraperitoneally administered with 50 μg of OVA (EndoGrade, Hyglos) 3 days after last sublingual administration and culled after 9 days of the OVA challenge. Splenocytes were isolated following conventional protocols, labelled with CFSE and stimulated in vitro with MV130 (10^7^ bact./mL), OVA (50 μg/mL) or control. Proliferation of CFSE‐labelled CD4^+^ T cells was monitored by flow cytometry after 5 days and cytokine production by Multiplex Cytometric kits (eBioscience) after 48 h of stimulation.

### Statistics

All data represent mean ± s.e.m. Unpaired or paired *t‐*test for differences between groups or Wilcoxon test when normality of data could not be inferred were performed with GraphPad Prism software, version 6.0. Significance was defined as *p < 0.05, **p < 0.01 and ***p < 0.001.

## Conflict of interest

J.L.S. is the founder and shareholder of Inmunotek SL. The rest of the authors declare no financial or commercial conflict of interest.

**Abbreviations**
CT: Cycle threshold
Ctrl: Control containing all excipients without bacteria
DCs: Dendritic cells
EF1α: Elongation factor 1α
GO: Gene ontology
hmoDCs: Human monocyte‐derived DCs
JAK‐STAT: Janus kinase‐Signal transducer and activator of transcription
mDCs: Myeloid DCs
NLRs: Nucleotide‐binding oligomerization domain‐like receptors
PBP: Polybacterial preparation
PMA: Phorbol 12‐myristate 13‐acetate
pDCs: Plasmacytoid DCs
RIPK2: Receptor‐interacting serine/threonine‐protein kinase 2
RRTIs: Recurrent respiratory tract infections
Syk: Spleen tyrosine kinase
SOCS: Suppressor of cytokine signalling

## Supporting information


**Supporting Information Figure 1**.MessengerRNAexpressionlevelsofIL‐12p40,IL‐12p35,TNF‐α,IL‐6,IL‐1β,IL‐23,andIL‐10genesinhmoDCstreatedwithMV130for0,1,3,6and24h.
**Supporting Information Figure 2**.ThegraphsdisplaythepercentageofinhibitionofTNF‐α,IL‐6,IL‐1β,IL‐23andIL‐10productionbyMV130orMV140‐treatedhmoDCsbytheinhibitorPepinh‐MYDrelatedtovehiclecontrols(Pepinh‐Control).Resultsarethemean ± s.e.m.(n = 6‐8).Unpaired*t*test*P < 0.05,**P < 0.01.

Peer review correspondence
