# Supplementary material for: Human dendritic cells activated with MV130 induce Th1, Th17 and IL‐10 responses via RIPK2 and MyD88 signalling pathways
Source: Eur J Immunol. 2017 Sep 14;48(1):180–93. doi: 10.1002/eji.201747024 (PMC5813220; doi:10.1002/eji.201747024)
Supplement: Supplementary file 1 — Supporting Information Figure 1.MessengerRNAexpressionlevelsofIL‐12p40,IL‐12p35,TNF‐α,IL‐6,IL‐1β,IL‐23,andIL‐10genesinhmoDCstreatedwithMV130for0,1,3,6and24h. Supporting Information Figure 2.ThegraphsdisplaythepercentageofinhibitionofTNF‐α,IL‐6,IL‐1β,IL‐23andIL‐10productionbyMV130orMV140‐treatedhmoDCsbytheinhibitorPepinh‐MYDrelatedtovehiclecontrols(Pepinh‐Control).Resultsarethemean ± s.e.m.(n = 6‐8).Unpairedttest*P < 0.05,**P < 0.01. [file EJI-48-180-s001.pdf]

European Journal of Immunology

Supporting Information
for
DOI 10.1002/eji.201747024

Cristina Cirauqui, Cristina Benito-Villalvilla, Silvia Sánchez-Ramón, Sofía Sirvent, Carmen M. Diez-Rivero, Laura Conejero, Paola Brandi, Lourdes Hernández-Cillero, Juliana Lucía Ochoa, Beatriz Pérez-Villamil, David Sancho, José Luis Subiza and Oscar Palomares

Human dendritic cells activated with MV130 induce Th1, Th17 and IL-10 responses via RIPK2 and MyD88 signalling pathways

© 2017 The Authors. *European Journal of Immunology* published by WILEY-VCH Verlag GmbH & Co. KGaA, Weinheim.

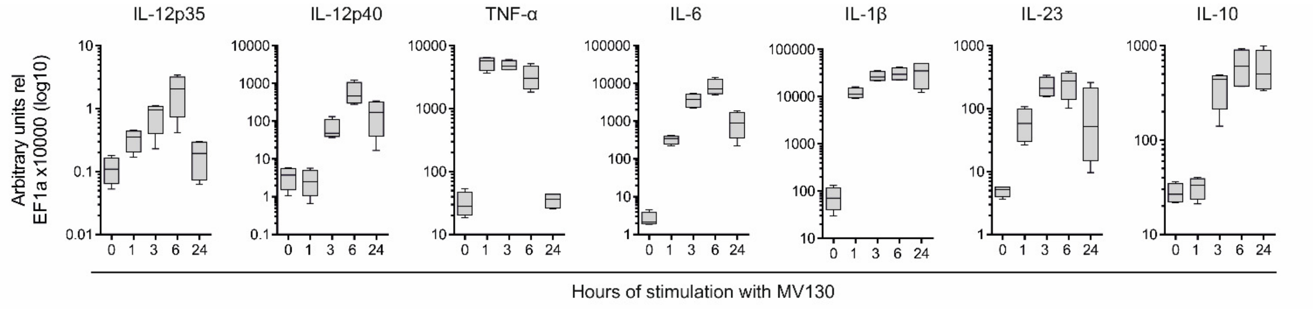

Cirauqi *et al*. Supplementary Figure 1

Supplementary Figure 1. Messenger RNA expression levels of IL-12p40, IL-12p35, TNF-α, IL-6, IL-1β, IL-23, and IL-10 genes in hmoDCs treated with MV130 for 0, 1, 3, 6 and 24 hours. Arbitrary units relative to EF1α are 2-ΔCT values multiplied by 10^4, with ΔCT defined as the difference between the cycle threshold value for the gene of interest and EF1α. Data are the mean ± s.e.m (n=4).

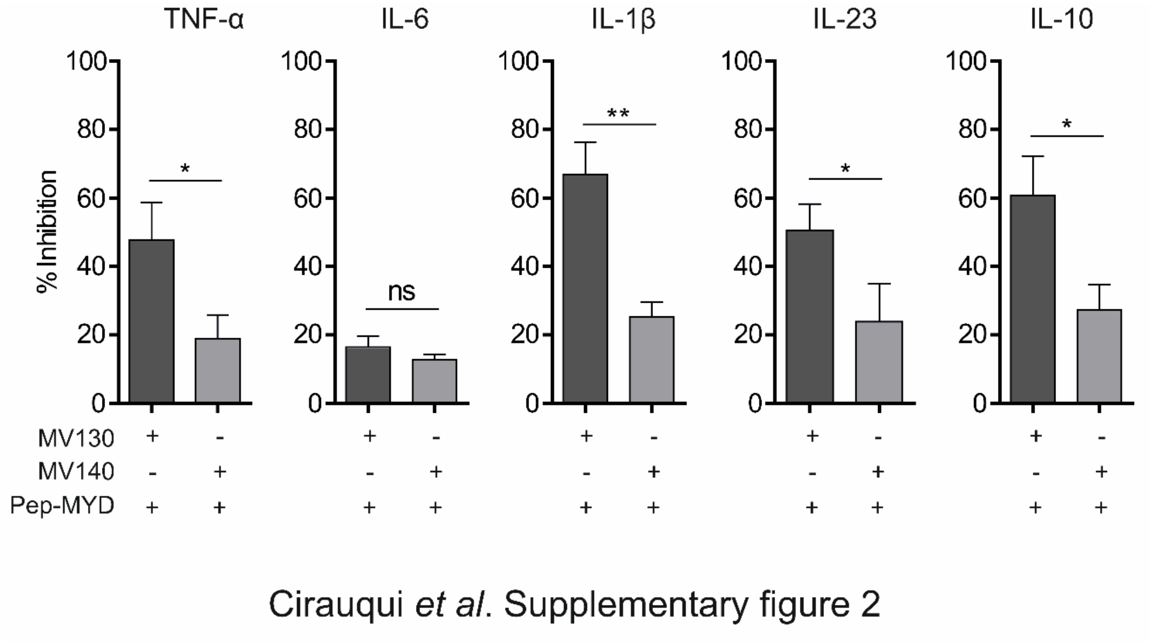

Supplementary Figure 2. The graphs display the percentage of inhibition of TNF-α, IL-6, IL-1β, IL-23 and IL-10 production by MV130 or MV140-treated hmoDCs by the inhibitor Pepinh-MYD related to vehicle controls (Pepinh-Control). Results are the mean± s.e.m. (n=6-8). Unpaired t test * $P < 0.05$, ** $P < 0.01$.
