# Supplementary material for: Human dendritic cells activated with MV130 induce Th1, Th17 and IL‐10 responses via RIPK2 and MyD88 signalling pathways
Source: Eur J Immunol. 2017 Sep 14;48(1):180–93. doi: 10.1002/eji.201747024 (PMC5813220; doi:10.1002/eji.201747024)
Supplement: Supplementary file 2 — Peer review correspondence [file EJI-48-180-s002.pdf]

European Journal of Immunology

Human dendritic cells activated with MV130 induce Th1, Th17 and IL-10 responses via RIPK2 and MyD88 signalling pathways

Cristina Cirauqui, Cristina Benito-Villalvilla, Silvia Sánchez-Ramón, Sofía Sirvent, Carmen M. Diez-Rivero, Laura Conejero, Paola Brandi, Lourdes Hernández-Cillero, Juliana Lucía Ochoa, Beatriz Pérez-Villamil, David Sancho, José Luis Subiza, Oscar Palomares

Correspondence: Dr. Oscar Palomares, Department of Biochemistry and Molecular Biology, School of Chemistry, Complutense University, Madrid, Spain

Review Timeline:	Submission date:	01-Mar-2017
	First Editorial decision:	27-Mar-2017
	Revision received:	14-Jul-2017
	Accepted:	08-Aug-2017

Handling Executive Committee member: Prof: Francesco Annunziato

Please note that the correspondence below does not include the standard editorial instructions regarding preparation and submission of revised manuscripts, only the scientific revisions requested and addressed.

First Editorial Decision
27-Mar-2017

Dear Dr. Palomares,

Manuscript ID eji.201747024 entitled "Human dendritic cells activated with MV130, a polybacterial preparation to treat recurrent respiratory tract infections, induce Th1, Th17 and IL-10 responses via RIPK2 and MyD88" which you submitted to the European Journal of Immunology has been reviewed. The comments of the referees are included at the bottom of this letter.

A revised version of your manuscript that takes into account the comments of the referees will be reconsidered for publication. Our Executive Editor encourages you to address all points raised and

perform the requested experiments. Should you disagree with any of the refereesâ€™ concerns, you should address this in your point-by-point response and provide solid scientific reasons for why you will not make the requested changes.

You should also pay close attention to the editorial comments included below. **In particular, please edit your figure legends to follow Journal standards as outlined in the editorial comments. Failure to do this will result in delays in the re-review process.**

Please note that submitting a revision of your manuscript does not guarantee eventual acceptance, and that your revision will be re-reviewed by the referees before a decision is rendered.

If the revision of the paper is expected to take more than three months, please inform the editorial office. Revisions taking longer than six months may be assessed by new referees to ensure the relevance and timeliness of the data.

Once again, thank you for submitting your manuscript to European Journal of Immunology and we look forward to receiving your revision.

Yours sincerely,
Nadja Bakocevic

On behalf of
Prof. Francesco Annunziato

Dr. Nadja Bakocevic
Editorial Office
European Journal of Immunology
e-mail: ejied@wiley.com
www.eji-journal.eu

****

Reviewer: 1

Comments to the Author
In Ciraqui et al., authors are studying immunomodulatory properties of MV130 heat-inactivated polybacterial preparation on human dendritic cells. First, they observe that MV130 induces potent

cytokine production by DCs (including TNF-a, IL-6, IL-1b, IL-23 and IL-10) and that this is dependent on RIPK2- and MyD88-mediated pathways. Second, they observe that MV130-stimulated DCs enhance IL-17, IFN-g and IL-10 secretion by CD4+ T cells in allogeneic mixed leukocyte reaction. Finally, they validate their findings in an in vivo model using BALB/c mice sublingually immunized with MV130.
Overall, this is a well performed study on the important subject of understanding mechanisms behind vaccine-induced antigen-independent immunity. However, the novelty of this work is diminished by rather general and predictable findings. A more detailed characterisation of induced pathways as well as studying possible interaction between them would increase the value of this study. Furthermore, in vivo experiments addressing antigen-independent function of the vaccine would substantially add to this work.
Major comments:
1) Fig. 1,2. Stimulation of DCs was performed with only one dose of MV130. The dose should be titrated, particularly when comparing DCs from healthy donors and patients, in order to be confident their high stimulatory dose does not mask differences.
2) Fig. 2 Co-secretion of IFN-g, IL-17 and IL-10 should be performed to further describe the phenotype and possible inflammatory potential of Th1/17 cells.
3) Fig. 6: No distinction between antigen-dependent and independent responses. The potential strength, and to some extent novelty, of this paper is addressing Ag-independent function of the MV130 vaccine. Using sublingual MV130 immunization followed by infection with an unrelated pathogen (e.g. RSV) or antigen (OVA/LPS) and assessing RSV or OVA-specific responses would be important (T cell proliferation, polarization, cytokine production upon restimulation as well as antibody levels and isotypes).

Reviewer: 2

Comments to the Author

General Comments:
This manuscript details a study examining DC and T cell immune responses induced by a whole heat-inactivated polybacterial preparation, i.e. MV130. The authors demonstrate that MV130 activates RIPK2 and MyD88 pathways of DCs and the activated DCs promote T cells to produce Th1/Th17 cytokines as well as IL-10. Furthermore, the authors speculate that modulation of DC function by MV130 contributes to the reduced rate of respiratory infections in RRTI patients after PBP treatment.

Specific Comments:
1. In the previously published report of the same research group (ref 50), the authors demonstrated that a polyvalent bacterial preparation MV140 promotes Th1/Th17 responses and IL-10 production, which is very similar to the function of MV130 described in this report. The phenomenon suggests that polyvalent bacterial preparations may play similar roles in activating innate receptors of DCs to promote T cell

activation. Therefore, it is necessary to use MV140 as a poly bacterial preparation control to describe specific functions of MV130 in modulating DC activation.

2. In Fig 1, the percentage of myeloid DCs in freshly isolated PBMCs is significantly lower in RRTI patients than in healthy subjects. What is the role of peripheral myeloid DCs in PBP treatment of recurrent respiratory tract infections? Furthermore, myeloid DCs, plasmacytoid DCs, and hmoDCs are different DC subtypes of distinct origin; do mDCs secret similar cytokines after MV130 stimulation as hmoDCs (shown in Fig 1B)?

3. in Fig 2A, the labeling of the X-axis should be CFSE? Furthermore, besides IL-5, IL-4 is an essential Th2 cytokine, the expression of IL-4 needs to be shown in Fig 2B as well.

4. pDCs are well known professional type I IFN producing cells. Therefore, it would be necessary to demonstrate the expression of type I IFN in Fig 3B to better understand pDC's function in the treatment of RRTI patients. Furthermore, although the percentage of myeloid DCs in PBMCs of RRTI patients is lower than that of healthy subjects, the cytokine production of T cells shows no difference after co-culture with total peripheral DCs from either RRTI patients or healthy subjects. The data in Fig 3C support the role of peripheral mDCs in PBP treatment and reduce the importance of the data shown in Fig 1A.

5. The role of IL-10 in the treatment of RRTIs patients with PBP remains vague. IL-10 is a well-known suppressive cytokine that downregulates immune reactions and thereby should favor recurrent infections of the patients. Elevated expression of IL-10 is observed in DCs stimulated with MV130/MV140/many other bacteria or in T cells co-cultured with the stimulated DCs, together with increased inflammatory cytokine expression. Does the production of IL-10 have a particular function in the treatment of RRTI patients, or is IL-10 production rather a common effect of PBP stimulation?

First Revision – authors' response
14-Jul-2017

Detailed point-by-point reply

The authors thank the editor and reviewers for their positive evaluation of this manuscript and for the useful comments and suggestions, which indeed helped to strength our previous results and to improve the quality of the paper. We have now addressed all the comments in detail by performing the requested new experiments and with proper discussions in the text.

The new data are now included in Figure 2D, Figure 4A/B/C/D, Figure 7 D/E/F, Supplementary Figure 1 and Supplementary Figure 2 of the revised manuscript. We have performed all the requested experiments. In particular, double intracellular staining for cytokine coexpression in the induced CD4$^+$ T cells (new Fig. 2D). We have also obtained enriched fractions of mDCs and pDCs to assess cytokine production after MV130 stimulation, including IFN-α in pDCs (new Fig. 4 A/B/C/D). We also tested the capacity of MV130 to promote Th1/Th17 and IL-10 responses against the unrelated antigen OVA in an *in vivo* mouse model (new Fig. 7 D/E/F). Supplementary figure 1 shows kinetics for the production of all the assayed cytokines in hmoDCs at the mRNA level and Supplementary figure 2 a direct comparison of MV130 and MV140 to highlight differences in the immunological mechanisms triggered by these polybacterial vaccines at the molecular level. The authors thank both reviewers for the positive evaluation and constructive comments, which indeed contributed to improve the quality of the manuscript.

Reviewer 1:

Major comments:

1."Fig. 1,2. Stimulation of DCs was performed with only one dose of MV130. The dose should be titrated, particularly when comparing DCs from healthy donors and patients, in order to be confident their high stimulatory dose does not mask differences."

"Author response"

We agree with the reviewer that this is a very important point. Actually, the dose used for DC activation (10^7 bact/mL) was selected based on initial dose-dependent titration experiments. As shown in Figure 1 (only for reviewer) we tested a wide range of doses for MV130 (ranging for 10^2 to 10^8 bact/mL). The production of proinflammatory cytokines (IL-6 and TNF-alpha) by DCs increased in a dose-dependent manner from 10^6 bact/mL, reaching maximum values at the dose of 10^8 bact/mL. In contrast, the production of IL-10 was optimal only at 10^7 bact/mL. Considering these data and the limitations of blood samples (especially from patients), we chose the 10^7 bact/mL as the most suitable dose to perform, at the same time, the comparisons for all the parameters displayed in Figures 1 and 2 and to assess potential differences between healthy donors and patients. We have now added a comment in the material and methods section of the

European Journal of Immunology

revised version of the manuscript indicating that the used dose was selected based on previous dose-dependent titration experiments as suggested by the reviewer (Page 17, line 374-6).

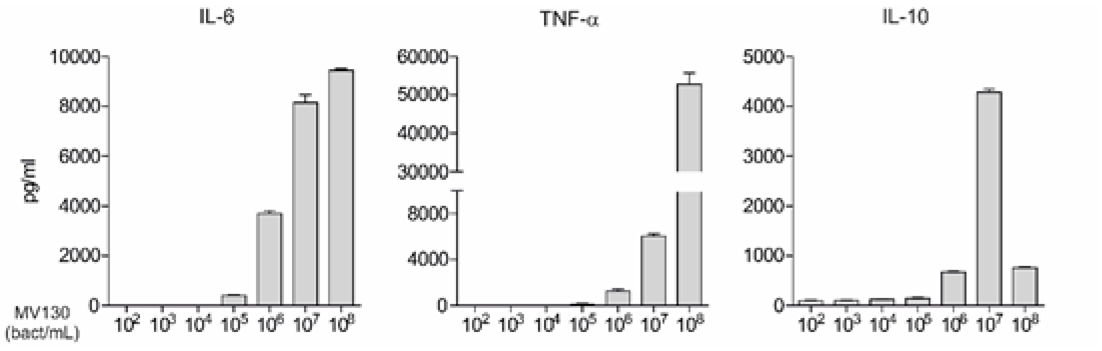

Figure 1: *Dose-dependent titration of MV130 in hmoDCs.* Cytokine levels in cell-free supernatants (IL-6, TNF-α, and IL-10) quantified by ELISA after stimulation of hmoDCs from healthy subjects (n=2) with MV130 at different concentrations for 24 hours.

2. "Fig. 2 Co-secretion of IFN-g, IL-17 and IL-10 should be performed to further describe the phenotype and possible inflammatory potential of Th1/17 cells."

"Author response"

We thank the reviewer for this interesting suggestion. We have characterized in more detail the phenotype of the T cells induced by MV130-treated hmoDCs as suggested. For that, we performed double intracellular staining experiments at the single cell level combining IFN-gamma or IL-17 with IL-10 antibodies and IFN-gamma with IL-17 antibodies (new Figure 2D in the revised manuscript). Our data showed that the IFN-γ or IL-17-producing T cells induced by MV130-treated hmoDCs did not simultaneously coexpress IL-10, suggesting that the induced IL-10-producing T cells could phenotypically correspond to type 1 regulatory T cells (Treg1). In contrast, we were able to detect a significantly higher population of T cells coexpressing IFN-γ and IL-17 induced by MV130-treated hmoDCs compared to control. Collectively, these data confirm the generation of Th1 and Th17 cells with possible inflammatory potential that could be regulated by the simultaneously induced IL-10-producing T cells.

We have included these new data in Figure 2D and properly discussed the results in the text (Results: Page 7, Lines 153-6 and Legend to Figure: Page 30, lines 727-729).

3. "Fig. 6: No distinction between antigen-dependent and independent responses. The potential strength, and to some extent novelty, of this paper is addressing Ag-independent function of the MV130 vaccine. Using sublingual MV130 immunization followed by infection with an unrelated pathogen (e.g. RSV) or antigen (OVA/LPS) and assessing RSV or OVA-specific responses would be important (T cell proliferation, polarization, cytokine production upon restimulation as well as antibody levels and isotypes)."

"Author response"

We thank the reviewer for raising this very interesting point and for the suggested experiments, which have contributed to improve the quality of the paper and to increase the novelty. We have performed the suggested experiments using sublingual MV130 or excipient control immunization and subsequent *in vivo* priming with an unrelated antigen (OVA) to assess *in vitro* OVA-specific responses as suggested. All the obtained results are now collected in the new Figure 7D/E/F of the revised manuscript.

Interestingly, our data demonstrated that MV130 sublingual immunization also significantly enhance immune responses against the unrelated antigen OVA. Our results showed that splenocytes from mice that were sublingually immunized with MV130 and subsequently challenged *in vivo* with OVA display significantly higher proliferation rates after *in vitro* stimulation with OVA than those from mice sublingually immunized with excipients as control. Remarkably, OVA-specific T cells from mice sublingually immunized with MV130 produced significantly higher levels of IFN-γ, IL-17 and IL-10 than those from the control group. We did not detect significant differences in OVA-specific IgG1 or IgG2a in any of the assayed conditions (data not shown). Collectively, these data indicate that MV130 vaccine is also able to promote enhanced immune responses against unrelated antigens, suggesting the potential capacity of this polybacterial preparation to confer protection not only against the components included in the vaccine but also against a broad range of different potential pathogens.

We have included these new data in Figure 7DEF and properly discussed the obtained results in the text (Abstract: Page 3, line 67; Results: Page 10-11, lines 241-253; Discussion: Page 12, line 260 and Page 13, line 306-309; Methods: Page 21, lines 487 and 491-494; Legend to figure (Page 33. Line 793-799).

Reviewer 2:

Specific Comments:

1. "In the previously published report of the same research group (ref 50), the authors demonstrated that a polyvalent bacterial preparation MV140 promotes Th1/Th17 responses and IL-10 production, which is very similar to the function of MV130 described in this report. The phenomenon suggests that polyvalent bacterial preparations may play similar roles in activating innate receptors of DCs to promote T cell activation. Therefore, it is necessary to use MV140 as a poly bacterial preparation control to describe specific functions of MV130 in modulating DC activation."

"Author response"

We thank the reviewer for this very interesting comment. As raised by the reviewer, we previously showed (ref 50) that MV140, a polybacterial preparation used for recurrent urinary tract infections and composed of 75% Gram negative and 25% Gram positive bacteria, also induce the generation of Th1/Th17 responses and IL-10 production as described in this paper for MV130 (90% Gram positive and 10% Gram negative bacteria). However, it is very important to keep in mind that the molecular mechanisms involved in the induction of these phenotypic and functional responses are different between MV130 and MV140. The effects induced by MV130 mainly depend on RIPK2 and MyD88, key adaptor molecules for NLRs and TLRs, respectively. In contrast, for MV140 the main involved pathways depend on Syk and MyD88 (CLRs and TLRs, respectively). Although TLRs contribute to the final outcomes in both cases, the relative contribution in MV130 and MV140 is also different. We have now included in the revised version of the manuscript new data showing these comparisons (Supplementary Figure 2 of the revised manuscript). These data show that the contribution of TLRs to the induction of all the assayed cytokines, except for IL-6, in human DCs was significantly higher for MV130 than MV140. Collectively, our data indicate that although the functional properties imprinted by different polybacterial preparations in human DCs seem to be similar, the underlying molecular mechanisms completely differ likely depending on the specific bacterial components included in each vaccine.

For most of the experiments performed to asses MV130 we also included MV140 as control. However, the detailed head to head comparison of MV130 and MV140 was beyond the scope of this manuscript and the inclusion of MV140 control data throughout the paper would generate very complex and larger figures that might generate confusion. Therefore, we have only included

European Journal of Immunology

in the revised version of the manuscript the data related to the relative contribution of TLRs in the production of cytokines by DCs after MV130 and MV140 stimulation (Supplementary Figure 2) and discussed in the text (Discussion: Page 14, lines 332-335; Legend to figure: Page 33, lines 808-811).

2. "In Fig 1, the percentage of myeloid DCs in freshly isolated PBMCs is significantly lower in RRTI patients than in healthy subjects. What is the role of peripheral myeloid DCs in PBP treatment of recurrent respiratory tract infections? Furthermore, myeloid DCs, plasmacytoid DCs, and hmoDCs are different DC subtypes of distinct origin; do mDCs secret similar cytokines after MV130 stimulation as hmoDCs (shown in Fig 1B)?"

"Author response"

We thank the reviewer for the comments and suggested experiments, which contributed to strengthen our results. Although the percentage of mDCs is significantly lower in RRTI patients than in healthy subjects (Fig. 1A), the isolated total DCs from healthy donors and patients equally respond to MV130 (Fig. 3B). These data suggest that mDCs from RRTI patients are functionally active and are able to respond to MV130 in a similar manner than mDCs from healthy donors. Following the reviewer's suggestion, we have obtained enriched fractions of mDCs and pDCs from healthy donors (Fig. 4A/C of the revised manuscript) to assess cytokine production after MV130 stimulation (Fig. 4B/D of the revised manuscript), including IFN-alpha responses in pDCs (Fig. 4E of the revised manuscript). As shown in the new Fig.4, mDCs produced higher levels of all the proinflammatory cytokines and IL-10 after MV130 stimulation than control, as previously shown for hmoDCs and total DCs (Fig. 1B and 3B, respectively). In contrast, pDCs only produced IL-6 after MV130 stimulation (Fig. 4D). Collectively, these data indicate that mDCs are the main responder DC subset after MV130 stimulation. Our results also suggest that although RRTI patients displayed lower percentages of mDCs than healthy donors in peripheral blood, they are functional and might well play a very important role in the MV130 treatment for RRTIs patients.

We have now included the new Fig.4 and properly discussed these data in the revised version of the manuscript (Results: Page 8, lines 168-178, Methods: Page 16-17, lines 370-373, Legend to Figure: Page 31, lines 743-751).

European Journal of Immunology

3. "In Fig 2A, the labeling of the X-axis should be CFSE? Furthermore, besides IL-5, IL-4 is an essential Th2 cytokine, the expression of IL-4 needs to be shown in Fig 2B as well."

"Author response"

We thank the reviewer for this comment and apologize for the previous mistake in Fig. 2A. The X-axis should be indeed CFSE and this has been corrected in the revised version of the manuscript.

We also measured IL-4 levels in the supernatants by ELISA and we did not detect production in any of the assayed conditions (Fig. 2, only for reviewer), likely due to the low half-life of this cytokine in culture supernatants. We did not include these negative results in the Figure 2B to keep the size and proportions of the Figure 2 homogeneous, but we have commented the results in the manuscript as data not shown (Results: Page 7, line 149-150).

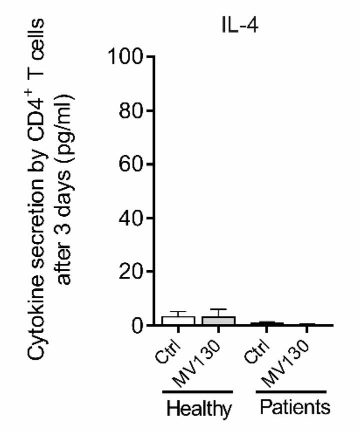

Figure 2. ELISA quantification of IL-4 cytokine in cell free supernatants produced by allogeneic naïve $CD4^+$ T cells primed by Ctrl- or MV130-activated hmoDCs from healthy subjects (n=8) and patients (n=6) after 3 days.

4."pDCs are well known professional type I IFN producing cells. Therefore, it would be necessary to demonstrate the expression of type I IFN in Fig 3B to better understand pDC's function in the treatment of RRTI patients. Furthermore, although the percentage of myeloid DCs in PBMCs of RRTI patients is lower than that of healthy subjects, the cytokine production of T cells shows no difference after co-culture with total peripheral DCs from either RRTI patients or healthy subjects. The data in Fig 3C support the role of peripheral mDCs in PBP treatment and reduce the importance of the data shown in Fig 1A.

European Journal of Immunology

“Author response”

We thank the reviewer for this interesting comment. As discussed in the point 2 of these reviewer’s comments, we have obtained an enriched fraction of mDCs and pDCs from healthy donors (Fig. 4 A/C of the revised manuscript) to assess cytokine production after MV130 stimulation (Fig. 4B/D/E of the revised manuscript). We measured the production of IFN-α by pDCs after MV130 stimulation as suggested by the reviewer (Fig. 4E). Enriched pDCs did not produced IFN-alpha after MV130 stimulation and IFN-α was detected only after stimulation with TLR9-L as positive control. Our data indicated that pDCs respond to MV130 by producing only IL-6 but none of the other cytokines assayed (Fig. 4D). In contrast, mDCs produced higher levels of all the proinflammatory cytokines and IL-10 after MV130 stimulation, as we previously showed for hmoDCs and total DCs (Fig. 1B and 3B, respectively). Collectively, these data indicate that mDCs are the main responder DC subset after MV130 stimulation. Supporting these data total DCs from RRTI patients induced similar T cell responses than those from healthy donors, indicating that mDCs are functionally active in both groups. Our results suggest that although RRTI patients displayed lower percentages of mDCs than healthy donors in peripheral blood, they might well play a very important role in the treatment of RRTIs with this polybacterial vaccine.

We have now included the new Fig.4 and properly discussed these data in the revised version of the manuscript (Results: Page 8, lines168-178, Methods: Page 16-17, lines 370-373, Legend to Figure: Page 31, lines 743-751).

5. “The role of IL-10 in the treatment of RRTIs patients with PBP remains vague. IL-10 is a well-known suppressive cytokine that downregulates immune reactions and thereby should favor recurrent infections of the patients. Elevated expression of IL-10 is observed in DCs stimulated with MV130/MV140/many other bacteria or in T cells co-cultured with the stimulated DCs, together with increased inflammatory cytokine expression. Does the production of IL-10 have a particular function in the treatment of RRTI patients, or is IL-10 production rather a common effect of PBP stimulation?”

“Author response”

We agree this is a very interesting point raised by the reviewer. To gain insight into the potential role of IL-10 in the MV130 treatment of RRTI patients, we supplemented our initial data with new experiments to assess the kinetics of IL-10 and the proinflammatory cytokines production at the

mRNA level in hmoDCs after MV130 stimulation (Supplementary Fig. 1 of the revised manuscript). Kinetic experiments revealed that IL-12p35/IL-12p40, TNF-α, IL-6, IL-1β, IL-23 and IL-10 mRNA expression levels were upregulated in MV130-activated hmoDCs in a time-dependent manner up to 6 hours. Interestingly, IL-10 mRNA levels were sustained after 24 hours. These data together with our IL-10 blocking experiments (Fig. 6C of the revised manuscript), suggest that IL-10 could be involved in the downregulation of the assayed pro-inflammatory cytokines. Collectively, our data revealed that MV130 immunomodulates human DCs in a dual way. MV130 promotes a rapid and potent pro-inflammatory response, inducing the secretion of cytokines to bias toward Th1/Th17 responses, which might contribute to enhance immune responses against intracellular and extracellular pathogens, respectively. On the other hand, MV130 also induces the production of high levels of IL-10 by DCs and the generation of IL-10-producing T cells, which is essential to avoid excessive deleterious responses, to enhance pathogen clearance and to keep tissue homeostasis. Further experiments to confirm all these aspects will be the aim of future investigations.

We have now included the new Supplementary Fig. 1 and properly discussed these data in the revised version of the manuscript (Results: Page 6, lines 134-139, Methods: Page 20-21, lines 466-483 and Legend to Figure: Page 33, lines 802-806).

Second Editorial Decision

03-Aug-2017

Dear Dr. Palomares,

It is a pleasure to provisionally accept your manuscript entitled "Human dendritic cells activated with MV130, a polybacterial preparation to treat recurrent respiratory tract infections, induce Th1, Th17 and IL-10 responses via RIPK2 and MyD88" for publication in the European Journal of Immunology. For final acceptance, please follow the instructions below and return the requested items as soon as possible as we cannot process your manuscript further until all items listed below are dealt with.

Please note that EJI articles are now published online a few days after final acceptance (see Accepted Articles: http://onlinelibrary.wiley.com/journal/10.1002/(ISSN)1521-4141/accepted). The files used for the

Accepted Articles are the final files and information supplied by you in Manuscript Central. You should therefore check that all the information (including author names) is correct as changes will NOT be permitted until the proofs stage.

We look forward to hearing from you and thank you for submitting your manuscript to the European Journal of Immunology.

Yours sincerely,
Marta Vuerich

on behalf of
Prof. Francesco Annunziato

Dr. Marta Vuerich
Editorial Office
European Journal of Immunology
e-mail: ejied@wiley.com
www.eji-journal.eu
